# Enhancing Edge Attack Strategy via an OWA Operator-Based Ensemble Design in Real-World Networks

**DOI:** 10.3390/e22080830

**Published:** 2020-07-29

**Authors:** Yuan Feng, Baoan Ren, Chengyi Zeng, Yuyuan Yang, Hongfu Liu

**Affiliations:** College of Intelligence Science and Technology, National University of Defense Technology, Changsha 410073, China; fengyuan18@nudt.edu.cn (Y.F.); renbaoan12@nudt.edu.cn (B.R.); zengchengyi08@nudt.edu.cn (C.Z.); yangyuyuan13@nudt.edu.cn (Y.Y.)

**Keywords:** network disintegration, OWA operator, edge attack strategy, structural similarity index

## Abstract

Network disintegration has been an important research hotspot in complex networks for a long time. From the perspective of node attack, researchers have devoted to this field and carried out numerous works. In contrast, the research on edge attack strategy is insufficient. This paper comprehensively evaluates the disintegration effect of each structural similarity index when they are applied to the weighted-edge attacks model. Experimental results show that the edge attack strategy based on a single similarity index will appear limited stability and adaptability. Thus, motivated by obtaining a stable disintegration effect, this paper designs an edge attack strategy based on the ordered weighted averaging (OWA) operator. Through final experimental results, we found that the edge attack strategy proposed in this paper not only achieves a more stable disintegration effect on eight real-world networks, but also significantly improves the disintegration effect when applied on a single network in comparison with the original similarity index.

## 1. Introduction

In our daily life, infrastructure networks such as the power system, aviation and transportation networks play an indispensable role in maintaining the normal operation of human society. For a long time, the academic community has been committed to improving the robustness of infrastructure networks in facing various potential threats. So far, the research on network robustness has achieved rich results [1,2,3,4,5]. Correspondingly, for terrorist networks [6] and disease spreading network [7] that are considered to be harmful, how to effectively destroy these networks has given birth to the study of network disintegration. The ultimate goal of studying the network disintegration is to obtain the optimal attack strategy to the nodes or edges in the network [8]. Unfortunately, the problem of finding the optimal set of key nodes or edges to destroy has been proved to be a non-deterministic polynomial-time (NP) hard problem [9,10]. Obviously, for the consideration of the calculation cost, it is more practical to find an effective attack strategy than the optimal one. An effective attack strategy should have both acceptable computational complexity and disintegration effect. The relative size of giant components is often used to evaluate the disintegration effect during the attack. The faster the size of giant components decreases, the more effective the attack strategy is.

Numerous studies have focused on the node-based attack strategy [11,12,13,14,15], the research on edge attack strategy only attracted a little attention. On the one hand, the edge of the network usually plays the role of transmitting information or load, and maintaining the connectivity of the network. The researchers used the load model of cascading failures to investigate the performance of the small-world network subject to deliberate attacks on node and edge. The results show that the edge attacks can result in larger cascading failures than node attacks [16]. On the other hand, in real-world networks, the node vulnerable to attack are often well protected, while the edge is a relatively easy target for attackers. For example, several representative blackout events are all triggered by line breakdown [17]. Based on the aforementioned two motivations, we believe that the edge attack strategy deserves a careful investigation. Researchers have launched concrete research on edge attack strategy based on edge importance. The importance of an edge can be quantified by the self-topological properties or the information of endpoints [18]. Considering the information such as the degree, the betweenness, and the harmonic closeness of nodes, Hao et al. proposed fourteen edge attack strategies [19]. Yin et al. adopted the edge betweenness as the weight of an edge, and explored the weighted-edge attack strategy with incomplete information [20]. Inspired by the weak tie phenomenon, the bridgeness index considers that the edges between larger cliques play a significant role in maintaining global connectivity [21].

Recently, based on the structural similarity index of link prediction, Qi et al. calculated the dissimilarity between the endpoints, which provided a new idea for finding the key edges [22]. For an NP-hard problem, the similarity index can give a heuristic solution with acceptable time consumption. However, the results of key edges identification vary greatly among networks with different structural characteristics [22], which may limit the stability and adaptability of the edge attack strategy based on the similarity index. As stated in He and Liu [23], ordered weighted averaging (OWA) operator is such a method which can effectively reduce the variance of similarity-based link prediction algorithm and improve the stability of link predictor. In order to select and organize these indices more reasonably, Wu et al. employed a typical metric precision to evaluate the performance of each index and filtered the indices with poor performance [24]. Simulations prove that the integrated learning and fusion algorithm are effective in solving the problem of link prediction, with the satisfactory effect [25]. Nevertheless, there is no relevant research on the combination of the OWA operator and edge attack strategy. Additionally, the effect of attacking the edges with different properties on network reliability is different [26]. Deng et al. have analyzed the nodes’ tendency of the optimal disintegration strategy [27]. Extending to the edge attack strategy, it is necessary to explore the relationship between the order of attacked edges and the disintegration effect.

In this paper, we synthetically evaluate the disintegration effect of 19 structural similarity indices under three representative edge attack sequences, revealing that only a few indices can achieve acceptable edge attack effects which can be divided into two categories according to their behaviors under different attack sequences. We further find that the stability and adaptability of the edge attack strategy based on a single index are very limited. Based on the experiments above, drawing on the idea of integrating similarity indices in link prediction, an edge attack strategy based on ordered weighted averaging (OWA) operator integration is proposed. The results on eight real-world networks suggest that compared with a single index, the edge attack strategy based on OWA operator integration uses the complementarity of different similarity indices to achieve a more stable and comprehensive disintegration effect. Additionally, we are surprised to find that by applying the OWA operator on a single network, the disintegration effect can be significantly better than any single index.

The article is organized as follows. In Section 2, under different attack sequences, we evaluate the disintegration effect of each structural similarity index when they are applied to the weighted-edge attacks model. In Section 3, we adopt the ordered weighted averaging operator to fuse the best performance indices on different networks, and propose an edge attack strategy based on OWA operator. In Section 4, an experimental comparison of the proposed edge attack strategy is conducted, and the reasons for its stability are analyzed. Finally, the conclusions are given in Section 5.

## 2. Evaluating the Performance of Structural Similarity Index Under Edge Attack Strategy

Before making the strategy of edge attack based on the OWA operator, we need to evaluate the performance of each similarity index in the preparation stage, and select the indices with excellent performance for fusion. There are 19 structural similarity indices that are considered in this paper. When we extend them to the level of edge attack strategy, we need to consider the impact of attack sequence and network structure on the performance of these indices.

### 2.1. Weighted-Edge Attacks Model Based on Structural Similarity Index

Complex networks can be presented by an undirected graph $G\left(V,E\right)$, in which *V* is the set of nodes, and *E* is the set of edges. Let N=|V| be the number of nodes and W=|E| be the number of edges, respectively. Denote by $A\left(G\right)={\left(a_{ij}\right)}_{N\times N}$ the adjacency matrix of *G*, in which $a_{ij}=a_{ji}=1$ once $v_{i}$ and $v_{j}$ are connected. Besides, let the diagonal matrix *D* be the degree matrix of *G*, where the diagonal elements correspond to the degrees of each vertex.

#### 2.1.1. Weight of Edge

Assume $e_{xy}$ is the edge connecting nodes *x* and *y*. According to the above discussion, in order to facilitate the test and selection of indices, we define the scores $s_{xy}$ of structural similarity indices as the weight of edge and record it as $w_{xy}$.

After the weight of the edge is determined, we will introduce three typical attack sequences based on the edge weight.

#### 2.1.2. Three Typical Weighted-Edge Attacks Sequences

With the decrease of the edge weight, the difference between the endpoints becomes larger. This difference may help to find the key edge in the network, but it also may not be suitable to measure the importance of the edge. Previous studies have shown that under several indices, removing the edges with lower weight may cause more serious damage to the connectivity of the network [22]. However, will they perform better in other attack sequences? Based on this problem, this paper considers the influence of the edge attack sequence. With the help of three different edge attack sequences, we find out the excellent performance indices and the corresponding attack sequence, which provides the basis for the subsequent development of an effective edge attack strategy.

We consider three attack sequences for weighted edges in the network, which are:

$\left(i\right)$ Highest Edge Weight First (HEWF): Edges are eliminated by the descending order of the weight $w_{xy}$, which means that the edge with larger weight will be removed preferentially.

$\left(\mathrm{ii}\right)$ Lowest Edge Weight First (LEWF): Edges are eliminated by the ascending order of the weight $w_{xy}$, which means that the edge with smaller weight will be removed preferentially.

$\left(\mathrm{iii}\right)$ Average Edge Weight First (AEWF): Edges near average weight will be eliminated preferentially. We denote the average weight $\langle w\rangle$. Let $w=\left(w_{1},w_{2},w_{3},\ldots w_{W}\right)$ be the weight sequence. Denoted by $\theta_{xy}=\left|w_{xy}-\langle w\rangle \right|$ the deviation value of the weight of edge $e_{xy}$ from the average weight. Therefore, edges near average weight will be removed preferentially, which means that edges are removed by the ascending order of $\theta_{xy}$.

#### 2.1.3. Evaluation Metrics

So far, we have defined the edge weight and the weighted-edge attack sequences. In general, if an index is effective, the network will disintegrate much faster when we remove the edges successively in one of the weighted-edge attacks sequences.

To visually observe the impact of removing edges, we can use the relative size of giant components to describe the process of network disintegration.

(1) $$\begin{array}{c} S=\frac{m}{N}\;, \end{array}$$

where *N* is the total number of nodes in the network, *m* is the size of the maximal connected component after removing a fraction of the edges. The more obvious the trend of decaying of the giant components as the edge is removed, the better the effect of using this index to attack the network.

To measure the disintegration effect in detail, we apply the measure *R*, which evaluates the size of the largest component during a target attack on edges [28]. As the edge deleting fraction *f* increases, the network will eventually collapse, until the size of the largest component reaches 1 (i.e., all nodes in the network are isolated nodes). The smaller the *R* is, the more efficient disintegration effect is. The definition of *R* is as follows:

(2) $$\begin{array}{c} R=\frac{1}{W}\sum_{f=1/W}^{1}S\left(f\right)\;, \end{array}$$

where *W* is the total number of edges and $S\left(f\right)$ is the relative size of giant component after removing fW largest edges. The normalization factor 1/W makes disintegration effect of networks with different sizes comparable.

This measure captures the network response to any fraction of edge remove. Apparently, if a structural similarity index is helpful to enhance edge attack, its *R* should be relatively small. We remark that some indices have large *R* no matter in which attack sequence, that is to say, they are invalid.

### 2.2. Index Performance Evaluation

This subsection describes the numerical simulations performed under three typical edge attack sequences to reveal the effectiveness of 19 structural similarity indices in promoting the network collapse. In these simulations, eight real-world networks are employed to verify the effectiveness of these indices.

See Appendix A for the definition of 19 structural similarity indices.

#### Experiment on Different Networks

All of the eight real-world networks are undirected and unweighted. For the case of multiple connected components, we only consider the largest one. Including Dolphins (friendship network) [29], Weaver (animal social network) [30], FWFB (food chain network) [31], Jazz (musician network) [32], C.elegans (neural network) [33], USAir (airline network) [34], NS (scientific collaboration network) [35] and Political blogs (hyper links between blogs) [36]. The basic properties of these systems are shown in Table 1.

**Figure 1:**
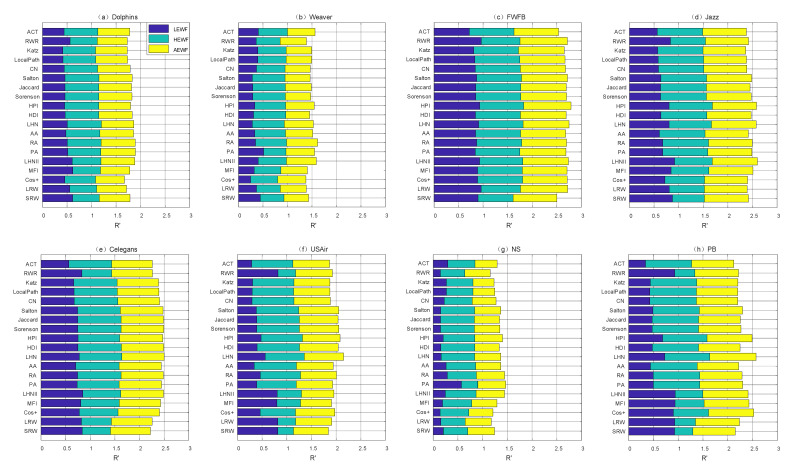
Comparison of the disintegration effect of 19 structural similarity indices under three typical attack sequences in eight real-world networks. The eight real-world networks are (**a**) Dolphins, (**b**) Weaver, (**c**) FWFB, (**d**) Jazz, (**e**) Celegans, (**f**) USAir, (**g**) NS and (**h**) PB. Three different color bars in the graph represent the R values of three edge attack sequences respectively. Because of $R\in \left(0,1\right)$, the upper bound of $R^{\prime }$ will not exceed 3.

Figure 1 shows the performance of each structural similarity index under different attack sequences on eight networks. The smaller value of the R, the more destructive the weighted-edge attack strategy based on the structural similarity index is. To facilitate the reader to visually compare the R values of indices under three attack sequences, we use a stacked bar plot to display.

The results indicate that not all similarity indices can significantly improve the effect of edge attack, which answers the question we raised in the previous content—whether all indices can effectively find the key edge in the network? The result is clearly that only a few individual indices have such capabilities.

**Figure 2:**
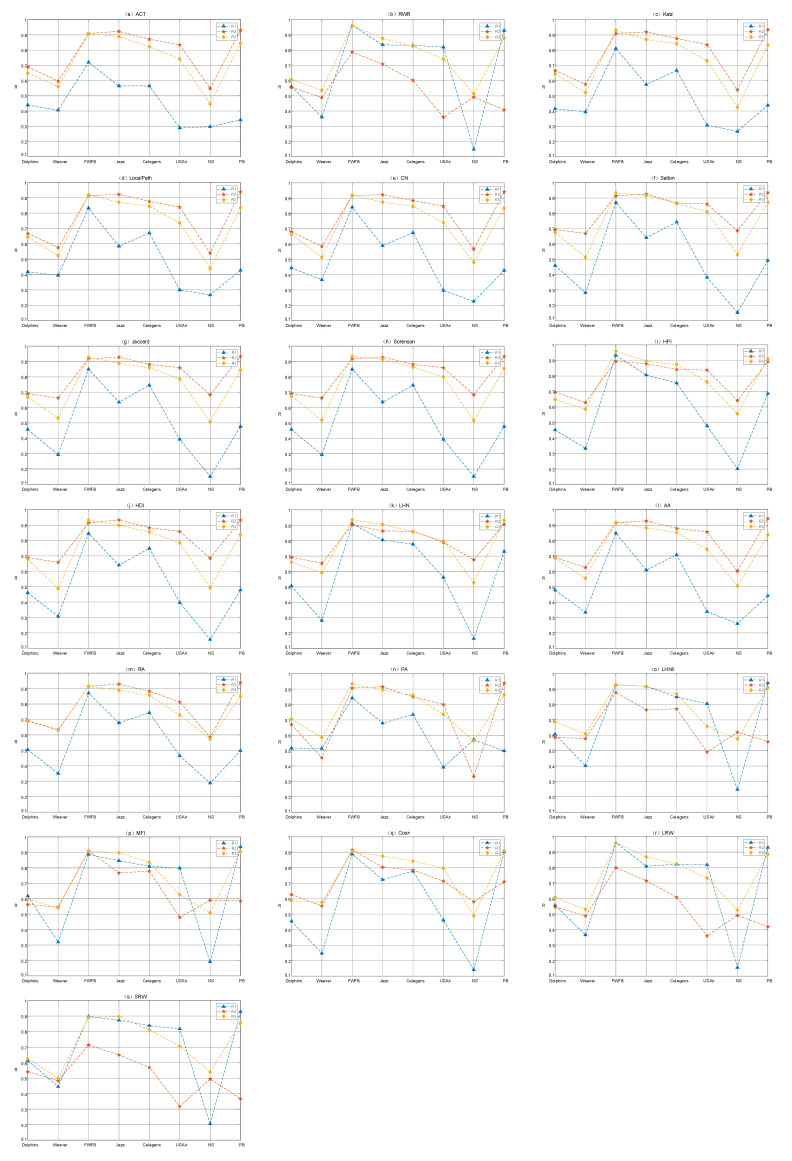
The R value of each similarity index under three attack sequences on eight networks. R1, R2 and R3 represent the R value under LEWF, HEWF and AEWF attack sequences respectively. (**a**–**s**) show the results of each similarity index respectively.

Moreover, the attack sequence will affect the behavior of the index. It can be found from Figure 2 that these indices can be roughly classified into two categories according to their behaviors. The indices such as Average Commute Time (ACT), Local Path, Katz, Leicht–Holme–Newman (LHN), Resource Allocation (RA) and Preferential Attachment (PA) tend to perform better under LEWF, corresponding to Figure 2a,c–n,p–q, indicating that when we attack the edge with a lower weight, the network connectivity will be more damaged. In some articles, this tendency is thought to be related to the “weak tie phenomenon” in social networks. For Random Walk with Restart (RWR), Local Random Walk (LRW), Superposed Random Walk (SRW) and Matrix Forest Index (MFI), which are classified as the second category, corresponding to Figure 2b,p,r,s, they have more advantages under HEWF. Out of our expectation, there are few indices that tend to choose the AEWF attack sequence, which is shown in Figure 2 that the value of R1 or R2 is always the smallest. The value of R3, which represents the AEWF attack sequence, is usually between R1 and R2.

As shown in Figure 2, LEWF has an absolute advantage on the NS and Weaver networks, which is reflected in that the minimum value of all indices except PA on these two networks is R1 (marked with a blue triangle). We notice that Leicht–Holme–Newman Index (LHNII), MFI, RWR, LRW, SRW exhibit abnormal behavior. Not only is the optimal attack sequence unexpectedly transformed from HEWF to LEWF (the value of the blue dotted line in Figure 2b drops sharply on NS and Weaver networks), but also the disintegration effect is better than ACT which has been performing well under LEWF (e.g., R1 values on NS and Weaver networks in Figure 2b are lower than those in Figure 2a). This transformation and the improvement of the disintegration effect may be related to the network structure properties.

To find out the reasons behind this abnormal behavior, we explore whether there is a certain relationship between the disintegration effect of edge attack strategy based on structural similarity index and network structure. In Table 1, in addition to several common network structure properties, we also consider a measure of network topology heterogeneity called the normalized entropy of the rank distribution. Wu et al. confirmed that this heterogeneity of topology is valuable for the study of invulnerability of complex networks [37]. For example, the heterogeneity leads to scale-free networks are robust against random attacks but fragile to intentional attacks. Therefore, quantitative measurement and analysis of the heterogeneity of network topology are of great significance to the study of the relationship between the disintegration effect and network structure.

The entropy of rank distribution and normalized entropy of rank distribution are defined as follows:

(3) $$\begin{array}{c} E_{Q}=-\sum_{r=1}^{N}Q\left(r\right)\ln Q\left(r\right)=\ln\left(\sum_{r=1}^{N}d_{r}\right)-\frac{\sum_{r=1}^{N}\left(d_{r}\ln d_{r}\right)}{\sum_{r=1}^{N}d_{r}}\;, \end{array}$$

(4) $$\begin{array}{c} NE_{Q}=\frac{E_{Q_{\max}}-E_{Q}}{E_{Q_{\max}}-E_{Q_{\min}}}=1-\frac{\ln\left[4\left(N-1\right)\right]/2-E_{Q}}{\ln\left[4\left(N-1\right)\right]/2-\ln N}\;. \end{array}$$

$Q\left(r\right)$ and $d_{r}$ are rank distribution and degree-rank function of graphs respectively. Please refer to the original paper for their definitions [37]. Obviously, the maximum value of $E_{Q}$ is $E_{Q_{\max}}=\ln N$ obtained for $d_{1}=d_{2}=\ldots =d_{N}$. Note that $d_{r}>0$ and the values of $d_{r}$ are all integers, so the minimum value of $E_{Q}$ is $E_{Q_{\min}}=\ln\left[4\left(N-1\right)\right]/2$ and occurs when $d_{1}=N-1$, $d_{2}=\ldots =d_{N}=1$.

We use Kendall’s tau correlation coefficient to determine the relationship between the 19 structural similarity indices’ disintegration effect R and the network structure properties. Kendall’s tau is a commonly used coefficient in mathematical statistics, mainly in measuring the statistical value of the correlation between two variables. From the previous analysis, we can see that the disintegration effect under LEWF is most obviously affected by the network structure, so we sort each index in descending order of its R value and get rank A. At the same time, according to rank A, the corresponding network structure properties are recorded in rank B.

For the pair of networks *i* and *j*, $a_{i}$ and $a_{j}$ in rank A are the R values of an index on this two networks, $b_{i}$ and $b_{j}$ in rank B are a structural property of this two networks. If both $a_{i}>a_{j}$ and $b_{i}>b_{j}$, or if both $a_{i}<a_{j}$ and $b_{i}<b_{j}$, then we think they are concordant, and discordant versa. The formula for Kendall’s tau is:

(5) $$\begin{array}{c} \tau =\frac{N_{1}-N_{2}}{\frac{1}{2}N\left(N-1\right)}\;, \end{array}$$

where $N_{1}$ is the number of concordant network pairs, $N_{2}$ is the number of discordant pairs, and *N* is the total number of the networks (eight networks are used in this paper). Obviously, the range of Kendall’s tau value is from −1 to 1. If the absolute value of Kendall’s tau between two ranks of a structural similarity index is larger, it means that the positive or negative correlation between the disintegration effect of this index and a network structural property is stronger. When Kendall’s tau = 0, there is no correlation between the two ranks and the disintegration effect of index is independent of the network structural property.

**Figure 3:**
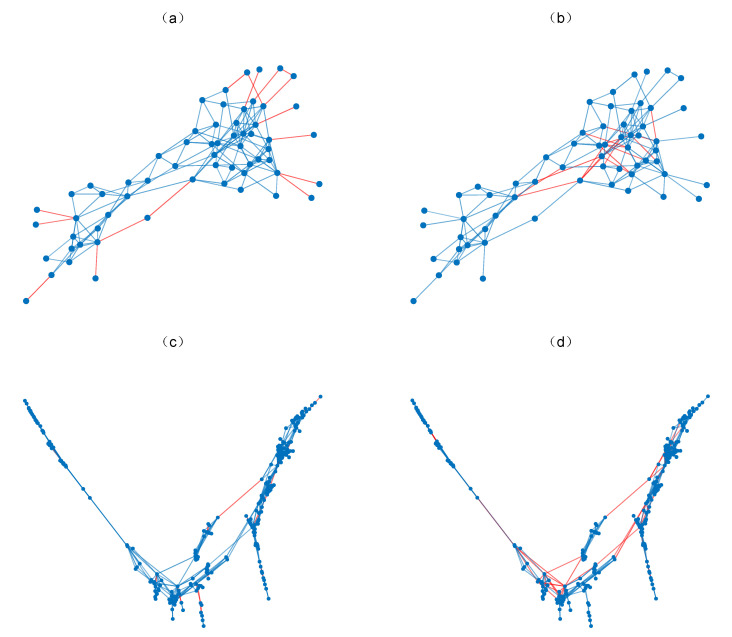
First 10% of the edges removed by edge attack strategy based on Average Commute Time (ACT) and Random Walk with Restart (RWR). (**a**,**c**) The edges removed preferentially by edge attack strategy based on ACT. (**b**,**d**) The edges removed preferentially by edge attack strategy based on RWR.

It can be seen from Table 2 that the disintegration effect of LHNII, MFI, RWR, LRW and SRW has a stronger positive correlation with the average degree, while a stronger negative correlation with the average shortest path length. This indicates that the R value of these indices are likely to decrease when the average degree gets smaller, or increase when the average shortest path length gets larger. The connection between the nodes in the network with a large average degree and small average shortest path length is closer, which may be the reason for the poor disintegration effect of these indices. Common Neighbors (CN), Local Path and Katz are defined based on the number of paths between two endpoints. These indices cannot effectively distinguish the key edges in the network when the clustering coefficient is small. Therefore, their disintegration effect is negatively related to the clustering coefficient. As for the ACT index based on average commuting time, unlike the above indices, it is more likely to be affected by the network heterogeneity. To sum up, the disintegration effect of the index largely depends on the network structure. A typical example is that under the LEWF attack sequence, the disintegration effect of ACT on the Dolphins network is much better than RWR, but on the NS network, the performance of RWR suddenly improves and surpasses ACT. In Figure 3, we mark the first 10% of the edges that are removed preferentially under the two indices in red, visualizing the impact of the network structure on the disintegration effect of the two indices.

From Figure 3, we can find that the damage degree of edge attack to network connectivity is related to network structure. ACT tends to remove the edge between external nodes and internal nodes, while RWR tends to remove the edge between internal nodes. For the Dolphins network, there are many redundant paths between the internal nodes. Removing a small number of edges between the internal nodes will not cause serious damage to the network connectivity. On the contrary, the external nodes with a small degree are more vulnerable. If the same proportion of edges are removed, ACT will produce more isolated nodes than RWR, thus causing greater damage to network connectivity. For the NS network, communities in the network are connected by a few edges. RWR can cause serious damage to global connectivity by removing these edges, while ACT ignores the edges between internal nodes. Therefore, the disintegration effect of RWR on NS network is much better than ACT.

In terms of definition, a single structural similarity index is like a detector only sensitive to the edge with a specific structure, so it is easy to find the edge with a similar structure in the network but ignore the edge with other structures. Suppose that the index is sensitive to the edge with structure A and the key edge which plays an important role in maintaining network connectivity has structure B. When A and B are similar, the index can quickly discover the edges with a similar structure to the key edge in the network thus improving the disintegration effect of edge attack. If there is a deviation between A and B, it is difficult to detect the key edge, resulting in a poor disintegration effect. We call the deviation between A and B as the “blind area” of the structural similarity index. If only a single index is used to develop the edge attack strategy, the “blind area” will limit the stability and adaptability of the disintegration effect.

## 3. Edge Attack Strategy Based on Ordered Weighted Averaging Operator

The goal of this paper is to obtain an edge attack strategy with stable disintegration effect under various network structures. In Section 2, we evaluate the disintegration effect of each structural similarity index when they are applied to the weighted-edge attacks model. The results show that most of the indices achieve the optimal disintegration effect under the edge attack sequence of Lowest Edge Weight First (LEWF). Moreover, we find that there is a “blind area” in the edge attack strategy based on a single index and limits the stability and adaptability of the disintegration effect.

Inspired by the idea of assembling similarity indices, this paper adopts the method of ordered weighted averaging (OWA) operator to fuse the indices with the best performance on different networks and proposes an edge attack strategy based on ordered weighted averaging operator. The underlying idea is to eliminate the influence of “blind area” by comprehensively considering the importance measurement of multiple indices to the edge. The combination of indices will produce more complementarity and a more stable disintegration effect.

### 3.1. OWA Operator

OWA operator was firstly proposed by Yager as a general information aggregation technology to aggregate multicriteria to form an overall decision function [38]. He et al. designed a link prediction integration algorithm based on OWA operator [23]. This algorithm fuses nine structural similarity indices based on local information, and solves the problem that local information-based algorithm often has poor stability in link prediction for the social network.

Given *n* individual similarity indices, the OWA operator is a mapping $F:R^{n}\to R$ with an associated weight vector $w=\left(w_{1},w_{2},\ldots ,w_{n}\right)$, which represents the weights of indices. The n-dimensional OWA operator is written as

(6) $$\begin{array}{c} F\left(a_{1},a_{2},\ldots ,a_{n}\right)=\sum_{i=1}^{n}w_{i}b_{i}\;, \end{array}$$

where $a_{i}$ denotes the score given by the *i*th index, $b_{i}$ is the *i*th value of $a_{1},a_{2},\ldots ,a_{n}$ according to some ranking criterion, and $w_{i}$ is the *i*th weight that represents the contribution of the corresponding individual index to a final result.

He et al.’s work demonstrated that O’Hagan’s maximum entropy method (MEM) [39] is an effective method to determine the weight vector of OWA operator. MEM is to solve the constrained nonlinear optimization model as

(7) $$\begin{array}{c} Maximize\;Disp\left(w\right)=-\sum_{}^{n}w_{i}\ln\left(w_{i}\right)\\ s.t.\;orness\left(w\right)=\alpha =\frac{1}{n-1}\sum_{}^{n}\left(n-i\right)w_{i}\\ \sum_{}^{n}w_{i}=1,\;w_{i}\in \left[0,1\right],\;i=1,2,\cdots ,n. \end{array}$$

where Disp(w) characterizes the extent to which all the indices are equally used in the aggregation process, while orness(w) measures the extent to which the aggregation is like an “or” operation. The weight vector can be adjusted by changing the optimism level factor α.

### 3.2. Edge Attack Strategy Based on OWA Operator

In order to make the edge attack strategy more effective, we have evaluated the performance of each index on promoting the disintegration effect in Section 2. LEWF is adopted as the edge attack sequence and the best performance indices on eight networks are selected to prepare for fusion, including ACT, Cos+ and Katz.

We need to standardize the structure similarity indices selected for fusion to be able to locate the weight of the edges in the interval [0,1]. Let $SN_{xy}^{ACT}$, $SN_{xy}^{Cos^{+}}$, $SN_{xy}^{Katz}$ be the normalizations of $S_{xy}^{ACT}$, $S_{xy}^{{\mathrm{Cos}}^{+}}$ and $S_{xy}^{Katz}$, the normalized structure similarity indices can be obtained through Equation (8):

(8) $$\begin{array}{c} SN_{xy}=\frac{S_{xy}-S_{xy\min}}{S_{xy\max}-S_{xy\min}}\;. \end{array}$$

Then, the edge weight of network is calculated by the structure similarity index ensemble method based on OWA operator. We denote the weight of edge connecting nodes x and y as $S_{xy}^{OWA}$, which is calculated as follows:

(9) $$\begin{array}{c} S_{xy}^{OWA}=\sum_{i=1}^{3}\left[w_{i}\cdot SN_{xy}^{\left(i\right)}\right]\;. \end{array}$$

$SN_{xy}^{ACT}$, $SN_{xy}^{Cos^{+}}$, and $SN_{xy}^{Katz}$ are arranged in descending order according to performance, and the performance of ACT is better than other two indices. We think it is reasonable that $SN_{xy}^{ACT}$ obtains the higher weight, because in Section 2, the R value of ACT is the smallest in most networks, which shows that ACT is reliable in judging the importance of edge in most cases, so it should get higher weight. For convenience, this approach is named ${\mathrm{EAS}}_{\mathrm{OWA}}$ (Edge attack strategy based on OWA operator) in this paper.

## 4. Experimental Comparison and Analysis

In this section, we test the disintegration effect of edge attack strategy based on OWA operator. The optimal weights of MEM corresponding to 13 different optimal level factors (i.e., α = 0.50, 0.60, 0.70, 0.80, 0.90, 0.91, 0.92, 0.93, 0.94, 0.95, 0.96, 0.97 and 0.98) are solved respectively by using LINGO software. The R value of ${\mathrm{EAS}}_{\mathrm{OWA}}$ with different associated weights being evaluated on eight networks. There is no particular reason for the selection of α, which is only used to check the impact of this parameter on the performance of ${\mathrm{EAS}}_{\mathrm{OWA}}$.

**Figure 4:**
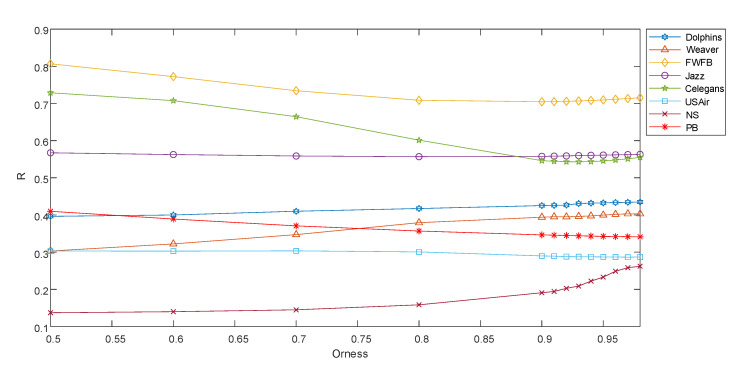
The disintegration effect of ${\mathrm{EAS}}_{\mathrm{OWA}}$ under different optimism level factor. Orness represents the value of optimistic level factor. The larger the value of Orness is, the larger is the first value of ordered weighted averaging (OWA) weight vector.

The results in Figure 4 show that the selection of parameter α has different effects on the performance of ${\mathrm{EAS}}_{\mathrm{OWA}}$ on eight networks. The larger the parameter α is, the more emphasis is placed on the individual index at the top of the ranking. With the increase of α, the R value of ${\mathrm{EAS}}_{\mathrm{OWA}}$ in Dolphins and Celegans networks decreased significantly, but it increased in NS and Weaver networks.

In order to verify the effectiveness of ${\mathrm{EAS}}_{\mathrm{OWA}}$, as a comparison, we introduce two commonly used methods to evaluate the importance of edge in maintaining network connectivity.

- Edge betweenness centrality [40]. Edge-betweenness centrality reflects the ratio of the number of shortest paths between pairs of nodes passing through the edge to the total number of shortest paths between all node pairs, defined as
  (10) $$\begin{array}{c} C_{E}=\sum_{s\neq t}\frac{\sigma_{st}\left(E\right)}{\sigma_{st}}\;, \end{array}$$
  where $\sigma_{st}$ is the number of shortest paths from node s to node t, and $\sigma_{st}\left(E\right)$ is the number of shortest paths from s to t that pass through edge E.
- Bridgeness [21]. Edges shared by dfferent communities are critical in connecting the network. Accordingly, the Bridgeness is defined as
  (11) $$\begin{array}{c} B_{E}=\frac{\sqrt{S_{x}S_{y}}}{S_{E}}\;, \end{array}$$
  where $S_{x}$ and $S_{y}$ are the clique sizes of nodes x and y, respectively. $S_{E}$ is the clique size of edge E. A fully connected subgraph with k nodes is called a clique of size k.

Note that unlike the LEWF edge attack sequence used in this paper, edges are removed in descending order of the weight under Edge betweenness centrality or Bridgeness. At the same time, the edge attack strategy based on the original structure similarity index is also considered to compare the performance of the OWA index and the original index.

**Figure 5:**
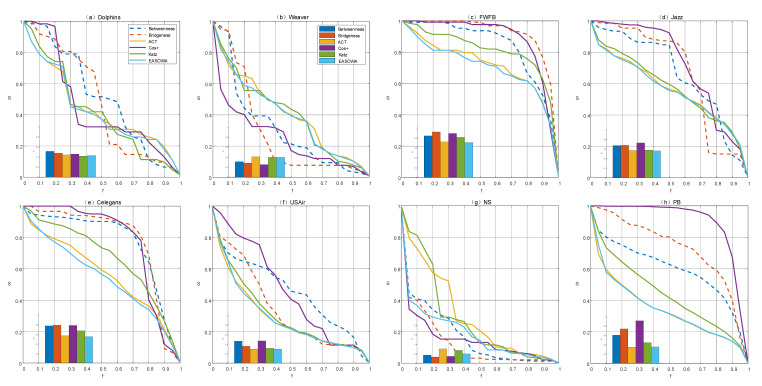
Comparison of the disintegration effects of six edge attack strategies. We choose the ${\mathrm{EAS}}_{\mathrm{OWA}}$ with α = 0.91. *f* is the fraction of removed edges in the network and *S* is the relative size of giant components. (**a**–**h**) correspond to the disintegration effects of six edge attack strategies on eight real-world networks, respectively.

Figure 5 shows the change curve of the relative size of giant components and the R values under six edge attack strategies.

Compared with the traditional methods, the edge attack strategy based on structural similarity index causes more damage to network connectivity. However, there is a serious disadvantage, which has been pointed out in Section 2, the single structural similarity index is limited by the “blind area” and the disintegration effect is instable. For example, the disintegration effect of ACT in Figure 5c–f,i is the best among the three structural similarity indices, but it suddenly becomes the worst in Figure 5b,g. Meanwhile, different “blind area” also bring complementarity between different indices. Cos+ has a very satisfactory disintegration effect in Figure 5b,g, and Katz performs better than ACT in Figure 5a.

Compared with a single index, the edge attack strategy based on OWA operator integration uses the complementarity of different similarity indices to achieve a more stable and comprehensive disintegration effect. Not only in Figure 5g, the disintegration effect of ${\mathrm{EAS}}_{\mathrm{OWA}}$ is greatly improved compared with ACT, but also in Figure 5c–f,i, ${\mathrm{EAS}}_{\mathrm{OWA}}$ still has the same optimal disintegration effect as ACT.

As a typical example of stabilizing the disintegration effect through complementarity among indices, in Figure 5b, where the disintegration effect of ACT and Cos+ is quite the opposite. The former is limited by “blind area”, which mistakenly judges ordinary edge as key edge, resulting in the maximum value of R. The latter can accurately find the key edge in the network, so the R value is the smallest.

**Figure 6:**
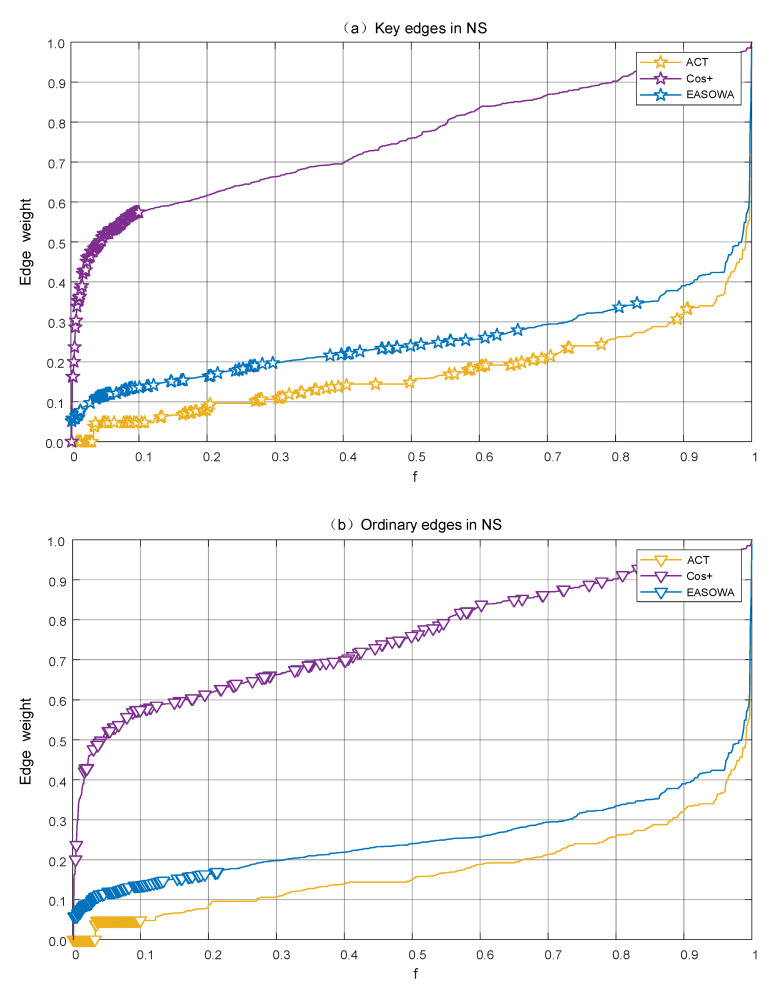
The positions of two types edge in Lowest Edge Weight First (LEWF) attack sequence in NS network. We choose the ${\mathrm{EAS}}_{\mathrm{OWA}}$ with α = 0.91. *f* is the fraction of removed edges in the network and LEWF attack sequence is to remove edges in ascending order of edge weight. (**a**) The positions of the key edges in LEWF attack sequence, represented by pentagram star; (**b**) the positions of the ordinary edges in LEWF attack sequence, represented by the inverted triangle.

Therefore, we assume that the first 10% of the edges in the LEWF attack sequence of Cos+ are the key edges, represented by pentagram star, and mark the corresponding positions of these edges in the attack sequence of ACT and ${\mathrm{EAS}}_{\mathrm{OWA}}$, as shown in Figure 6a. Similarly, the first 10% of the edges in the LEWF attack sequence of ACT are assumed to be ordinary edges, represented by inverted triangles, and the corresponding positions of these edges in the attack sequence of Cos+ and ${\mathrm{EAS}}_{\mathrm{OWA}}$ are marked out, as shown in Figure 6b.

In Figure 6a, the first 10% of the edges in the Cos+ attack sequence are all key edges, 91 in total, which will be removed, preferentially in the edge attack strategy. The result shows that ACT cannot recognize these key edges effectively. Only 25 key edges fall into the first 10% of the ACT attack sequence. With the same fraction *f*, the number of key edges removed by ACT is far less than Cos+, resulting in a larger R value. After the fusion of the OWA operator, the number of key edges within the first 10% of ${\mathrm{EAS}}_{\mathrm{OWA}}$ attack sequence is 45, which is 20 more than ACT. It is obvious from Figure 6a that the positions of key edges in ${\mathrm{EAS}}_{\mathrm{OWA}}$ not only move forward as a whole, but also distribute more intensively in the front segment.

In Figure 6b, the first 10% of the edges in the ACT attack sequence are ordinary edges, 91 in total, which are not as important as the key edges in maintaining network connectivity. If the ordinary edges occupy the priority removed position, the attack effect will be reduced. Most of the ordinary edges are assigned higher weights in Cos+, indicating that their structural features are quite different from the key edges. After OWA operator fusion, the number of ordinary edges in the first 10% of ${\mathrm{EAS}}_{\mathrm{OWA}}$ attack sequence is 65, which is 26 fewer than ACT. From Figure 6b, the positions of ordinary edges in ${\mathrm{EAS}}_{\mathrm{OWA}}$ move backward as a whole and partially move to a position other than 10%.

On the one hand, Cos+ is not limited by the “blind area” of ACT and can accurately identify key edges. On the other hand, Cos+ will correct the wrong judgment that ACT recognizes ordinary edges as key edges. Therefore, compared with ACT, the first 10% attack sequence of ${\mathrm{EAS}}_{\mathrm{OWA}}$ increased 20 key edges and reduced 26 ordinary edges, which stabilized the disintegration effect.

In addition to making the disintegration effect more stable, the result in Figure 5e shows that the disintegration effect of ${\mathrm{EAS}}_{\mathrm{OWA}}$ is further improved compared with the single index with the best disintegration effect. This unexpected improvement is also confirmed in Figure 5c,d. This phenomenon inspires us to explore whether the disintegration effect will be improved by using OWA operator to fuse the best performance indices on a single network. In fact, each index can be regarded as a classifier to distinguish the key edges from the ordinary edges. Through the combination of multiple effective classifiers, we can get better classification results. By controlling the weight vector of OWA operator, different combinations can be adjusted.

Throughout the paper, we mainly show the results of three representative networks: FWFB, Celegans and NS by figures as examples. The three indices with the minimum R value on each network and their attack sequences are listed as follows.

- FWFB: ACT (under LEWF), SRW (under HEWF) and RWR (under HEWF).
- Celegans: ACT (under LEWF), SRW (under HEWF) and RWR (under HEWF).
- NS: Cos+ (under LEWF), RWR (under LEWF) and Jaccard (under LEWF).

Based on the original OWA operator ensemble model, a new ensemble model is proposed to integrate the indices under different attack sequences.

(12) $$\begin{array}{c} S_{xy}^{OWA}=\sum_{i=1}^{3}\left[c_{i}\cdot w_{i}\cdot SN_{xy}^{\left(i\right)}\right]\\ \left\{\begin{array}{c} c_{i}=1,\;\;\;\;\;\;\mathrm{if}\;\;\mathrm{index}\;\;i\;\;\mathrm{is}\;\;\mathrm{under}\;\;\mathrm{LEWF}\\ c_{i}=-1,\;\;\mathrm{if}\;\;\mathrm{index}\;\;i\;\;\mathrm{is}\;\;\mathrm{under}\;\;\mathrm{HEWF} \end{array}\right.\;, \end{array}$$

where the coefficient $c_{i}$ represents the type of attack sequence. After multiplying the attack sequence under HEWF by the coefficient $c_{i}$, it can be converted into the attack sequence under LEWF.

**Figure 7:**
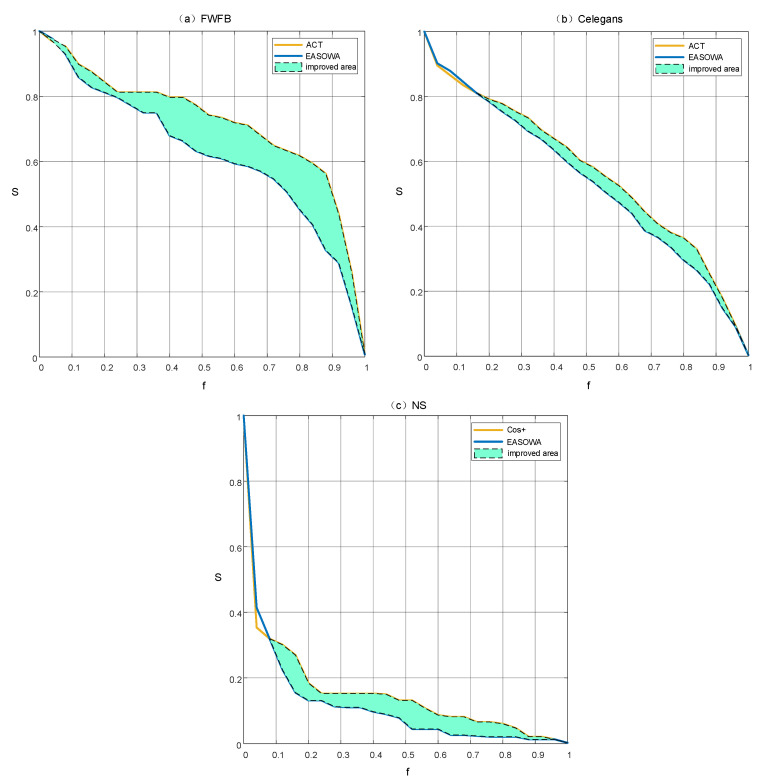
The disintegration effect of ${\mathrm{EAS}}_{\mathrm{OWA}}$ on three typical networks. To highlight the improvement, the figure adds the disintegration effect of the best similarity index on each network as a comparison. Areas covered in cyan represent the “improved area”. *f* is the fraction of removed edges in the network and *S* is the relative size of giant components. (**a**) The disintegration effect of ${\mathrm{EAS}}_{\mathrm{OWA}}$ with α = 0.6 on FWFB network; (**b**) The disintegration effect of ${\mathrm{EAS}}_{\mathrm{OWA}}$ with α = 0.6 on Celegans network; (**c**) The disintegration effect of ${\mathrm{EAS}}_{\mathrm{OWA}}$ with α = 0.5 on NS network.

The results in Figure 7 show that the edge attack strategy based on OWA operator integration can improve the effect of disintegration on a single network. We find that the curves of ${\mathrm{EAS}}_{\mathrm{OWA}}$ declines faster than a single similarity index, especially in the two networks of FWFB and NS. The area between two curves in each subgraph demonstrates the improvement of the effect of network disintegration due to the OWA operator.

## 5. Conclusions

From the perspective of edge attack, this paper systematically studies the performance of 19 structural similarity indices on the real-world network, taking into account the impact of attack sequence and network structure on the disintegration effect of the index. The results show that the stability and generalization ability of edge attack strategy based on a single index are poor. On this basis, we design an edge attack strategy based on ordered weighted average (OWA) operator integration. Experimental results on eight real-world networks validate that our method not only obtains better stability and adaptability, but also can be used to improve the disintegration effect on a single network.

At present, the research on the disintegration strategy of complex networks has been extended to multi-layer networks, heterogeneous networks and interdependent networks. Next, we will study the edge attack strategy on the above networks in our future work.

In addition, the work of our paper is from the perspective of the attacker, without considering potential opponent information. In fact, opponents may adopt corresponding defense strategies. Therefore, in future work, we need to use game theory to provide a suitable framework for modeling the confrontations between the attacker and defender [41,42,43,44,45].

## Figures and Tables

**Table 1:** The structure properties and normalized entropy of rank distribution values of eight networks. Structure properties include network size *N*, edge number *E*, average degree $\langle k\rangle$, average shortest path length $\langle d\rangle$, assortative coefficient *r*, clustering coefficient *C* and normalized entropy of rank distribution $NE_{Q}$.

Network	*N*	*E*	$\langle k\rangle$	$\langle d\rangle$	r	C	${\mathrm{NE}}_{Q}$
Dolphins	62	159	5.13	3.36	−0.04	0.30	0.1275
Weaver	117	304	5.19	4.46	0.06	0.66	0.1412
FWFB	128	2075	32.42	1.78	−0.11	0.33	0.0633
Jazz	198	2742	27.70	2.24	0.02	0.63	0.1041
C.elegans	297	2148	14.46	2.46	−0.16	0.31	0.1296
USAir	332	2126	12.81	2.74	−0.21	0.75	0.3528
NS	379	914	4.82	6.04	−0.08	0.80	0.1083
PB	1222	16714	27.36	2.74	−0.22	0.36	0.2418

**Table 2:** Kendall’s tau correlation coefficient between the disintegration effect of 19 structural similarity indices and network structure properties. The symbol of the number in the table indicates positive and negative correlation, and the value indicates correlation strength. Bold italics represent the disintegration effect of structural similarity index has a strong correlation with the network structure property.

	ACT	RWR	Katz	LocalPath	CN	Salton	Jaccard	Sørenson	HPI	
**$\langle k\rangle$**	0.3889	**0.8889**	0.5000	0.5000	0.4444	**0.6111**	**0.6111**	**0.6111**	**0.7778**	
**$\langle d\rangle$**	−0.4444	**−0.8333**	−0.5556	−0.5556	−0.5000	**−0.6667**	**−0.6667**	**−0.6667**	**−0.8333**	
***r***	0.1111	−0.3889	−0.1111	−0.1111	−0.0556	−0.2222	−0.2222	−0.2222	−0.1667	
***C***	−0.5000	−0.2222	**−0.6111**	**−0.6111**	**−0.6667**	−0.5000	−0.5000	−0.5000	−0.3333	
**$NE_{Q}$**	**−0.6111**	−0.1111	−0.3889	−0.3889	−0.4444	−0.2778	−0.2778	−0.2778	−0.3333	
	**HDI**	**LHN**	**AA**	**RA**	**PA**	**LHNII**	**MFI**	**Cos+**	**LRW**	**SRW**
**$\langle k\rangle$**	**0.6111**	**0.8333**	0.5556	0.5556	0.2222	**0.8333**	**0.8333**	**0.6667**	**0.7778**	**0.8333**
**$\langle d\rangle$**	**−0.6667**	**−0.8889**	**−0.6111**	**−0.6111**	−0.2778	**−0.7778**	**−0.7778**	**−0.6111**	**−0.7222**	**−0.7778**
***r***	−0.2222	−0.2222	−0.1667	−0.1667	−0.0556	−0.4444	−0.4444	−0.5000	−0.5000	−0.4444
***C***	−0.5000	−0.2778	−0.5556	−0.5556	−0.3333	−0.1667	−0.1667	−0.3333	−0.2222	−0.1667
**$NE_{Q}$**	−0.2778	−0.2778	−0.3333	−0.3333	**−0.6667**	−0.0556	−0.0556	0.0000	0.0000	−0.0556

## Appendix A

In this paper, the structural similarity indices we used to define the edge weight $w_{xy}$ are classified into three categories: local similarity indices, global indices and the quasi-local indices.

Local similarity indices:

(1) Common Neighbors (CN). For a node *x*, let $\Gamma \left(x\right)$ denote the set of neighbors of *x*. The similarity of two nodes *x* and *y* is defined as the number of neighborhood overlap which can be simply calculated as

$$S_{xy}^{CN}=\left|\Gamma \left(x\right)\cap \Gamma \left(y\right)\right|$$

(2) Salton Index [46]. Let $k_{x}$ denote the degree of node *x*. The index is defined as

$$S_{xy}^{Salton}=\frac{|\Gamma \left(x\right)\cap \Gamma \left(y\right)|}{\sqrt{k_{x}k_{y}}}$$

(3) Jaccard Index [47]. More than 100 years ago, Jaccard proposed this index, which is defined as

$$S_{xy}^{Jaccard}=\frac{|\Gamma \left(x\right)\cap \Gamma \left(y\right)|}{|\Gamma \left(x\right)\cup \Gamma \left(y\right)|}$$

(4) Sørensen Index [48]. It is mainly applied to ecological data research. The formula is as follows

$$S_{xy}^{Srensen}=\frac{2|\Gamma \left(x\right)\cap \Gamma \left(y\right)|}{k_{x}+k_{y}}$$

(5) Hub Promoted Index (HPI) [49]. It is used to quantitatively describe the topological similarity of each pair of reactants in metabolic networks, defined as

$$S_{xy}^{HPI}=\frac{|\Gamma \left(x\right)\cap \Gamma \left(y\right)|}{\min\left\{k_{x},k_{y}\right\}}$$

(6) Hub Depressed Index (HDI). Similar to the HPI index, except that the denominator takes the maximum degree value of the two endpoints, and is defined as

$$S_{xy}^{HDI}=\frac{|\Gamma \left(x\right)\cap \Gamma \left(y\right)|}{\max\left\{k_{x},k_{y}\right\}}$$

(7) Leicht–Holme–Newman Index (LHNI) [50]. It is defined as

$$S_{xy}^{LHNI}=\frac{|\Gamma \left(x\right)\cap \Gamma \left(y\right)|}{k_{x}k_{y}}$$

where it assigns higher similarity to node pairs that have more common neighbors.

(8) Preferential Attachment Index (PA). The probability of a new connection between nodes *x* and *y* is proportional to the product of $k_{x}$ and $k_{y}$.

$$S_{xy}^{PA}=k_{x}k_{y}$$

(9) Adamic–Adar Index (AA) [51]. The idea of this index is that the contribution of less connected neighbors is greater than more connected neighbors, so the former should be assigned higher weight, and is defined as

$$S_{xy}^{AA}=\sum_{z\in \Gamma \left(x\right)\cap \Gamma \left(y\right)}\frac{1}{\log\;\;k_{z}}$$

(10) Resource Allocation Index (RA) [52]. This index is inspired by the network resource allocation process [53], which is defined as

$$S_{xy}^{RA}=\sum_{z\in \Gamma \left(x\right)\cap \Gamma \left(y\right)}\frac{1}{k_{z}}$$

Global similarity indices:

(11) Katz Index [54]. This index considers all paths of the network, and the contribution of higher-order paths becomes smaller as the parameter β decreases and is defined as

$$S_{xy}^{Katz}=\sum_{l=1}^{\infty }\beta^{l}\cdot \left|paths_{xy}^{\langle l\rangle }\right|=\beta A_{xy}+\beta^{2}{\left(A^{2}\right)}_{xy}+\beta^{3}{\left(A^{3}\right)}_{xy}+\cdots$$

(12) Leicht–Holme–Newman Index (LHN-II) [50]. This index is defined as

$$S_{xy}^{LHN-II}=\phi AS+\psi I=\psi {\left(I-\phi A\right)}^{-1}=\psi \left(I+\phi A+\phi^{2}A^{2}+\cdots \right),$$

where ϕ and ψ are adjustable parameters.

(13) Average Commute Time (ACT). This index measures the similarity of each pair of nodes by the average number of steps that random walk particles starting from node *x* to node *y*. This index is defined as

$$S_{xy}^{ACT}=\frac{1}{l_{xx}^{+}+l_{yy}^{+}-2l_{xy}^{+}},$$

where $l_{xy}^{+}$ represents the element corresponding to the position of the *x*-th row and *y*-th column in the matrix $L^{+}$. The matrix $L^{+}$ can be obtained in terms of the pseudoinverse of the Laplacian matrix, $L\left(L=D-A\right)$.

(14) Random Walk with Restart (RWR). It assumes that random walk particles return to the initial position with a certain probability at each step. The RWR index is thus defined as

$$S_{xy}^{RWR}=q_{xy}+q_{yx},$$

where $q_{xy}$ represents the probability this random walker locates at node *y* in the steady state.

(15) Cosine based on $L^{+}$ (Cos+) [55]. This index is an inner-product-based measure. In the Euclidean space spanned by $v_{x}=\Lambda^{\frac{1}{2}}U^{T}{\vec{e}}_{x}$, where *U* is an orthonormal matrix made of the eigenvectors of $L^{+}$ ordered in the decreasing order of the corresponding eigenvalue $\lambda_{x}$, $\Lambda =\mathrm{diag}\left(\lambda_{x}\right)$, ${\vec{e}}_{x}$ is an N×1 vector with the *x*-th element equal to 1 and others all equal to 0, and *T* is the matrix transposition, the pseudoinverse of the Laplacian matrix are the inner products of the node vectors, $l_{xy}^{+}=v_{x}^{T}v_{y}$. Accordingly, the cosine similarity is defined as the cosine of the node vectors.

$$S_{xy}^{{\mathrm{Cos}}^{+}}=\cos{\left(x,y\right)}^{+}=\frac{v_{x}^{T}v_{y}}{|v_{x}\left|\cdot \right|v_{y}|}=\frac{l_{xy}^{+}}{\sqrt{l_{xx}^{+}\cdot l_{yy}^{+}}}.$$

(16) Matrix Forest Index (MFI) [56]. Based on the matrix forest theory, this index is defined as

$$S={\left(I+\alpha \cdot L\right)}^{-1},\;\;\;\;\alpha >0.$$

Quasi-local indices:

(17) Local Path Index (LP) [52,57]. In order to make the distinction between node pairs more obvious, the LP index further considers the third-order paths on the basis of the Common Neighbors.

$$S^{LP}=A^{2}+\alpha A^{3},$$

where α is an adjustable parameter and *A* is adjacency matrix of the network. Clearly, this index degenerates to CN when α=0.

(18) Local Random Walk (LRW) [58]. To measure the similarity between nodes *x* and *y*, a random walker is initially put on node x and thus the initial density vector ${\vec{\pi }}_{x}\left(0\right)={\vec{e}}_{x}$. This density vector evolves as ${\vec{\pi }}_{x}\left(t+1\right)=P^{T}{\vec{\pi }}_{x}\left(t\right)$ for t≥0. The LRW index at time step *t* is thus defined as

$$S_{xy}^{LRW}\left(t\right)=q_{x}\pi_{xy}\left(t\right)+q_{y}\pi_{yx}\left(t\right),$$

where *q* is the initial configuration function.

(19) Superposed Random Walk (SRW) [58]. It is defined as

$$S_{xy}^{SRW}\left(t\right)=\sum_{\tau =1}^{t}S_{xy}^{SRW}\left(\tau \right)=\sum_{\tau =1}^{t}\left[q_{x}\pi_{xy}\left(\tau \right)+q_{y}\pi_{yx}\left(\tau \right)\right].$$

On the basis of LRW, the value of SRW is obtained by adding the step *t* and its previous result. This index gives the neighboring nodes more chance to connect to the target node, taking full account of the local characteristics of many real network connections.
